# 565 Rehabilitation Time to Evaluation Report: A Quality Improvement Study

**DOI:** 10.1093/jbcr/irad045.161

**Published:** 2023-05-15

**Authors:** Scott Vocke, Emily Werthman, Julie Caffrey, Brooke Dean, Joshua Rodriguez, Gregory Andre

**Affiliations:** Johns Hopkins Bayview Medical Center, Baltimore, Maryland; Johns Hopkins Bayview Medical Center, Baltimore, Maryland; Johns Hopkins University School of Medicine, Baltimore, Maryland; Johns Hopkins Bayview Medical Center, Baltimore, Maryland; Johns Hopkins Bayview Medical Center, Baltimore, Maryland; Johns Hopkins Bayview Medical Center, Baltimore, Maryland; Johns Hopkins Bayview Medical Center, Baltimore, Maryland; Johns Hopkins Bayview Medical Center, Baltimore, Maryland; Johns Hopkins University School of Medicine, Baltimore, Maryland; Johns Hopkins Bayview Medical Center, Baltimore, Maryland; Johns Hopkins Bayview Medical Center, Baltimore, Maryland; Johns Hopkins Bayview Medical Center, Baltimore, Maryland; Johns Hopkins Bayview Medical Center, Baltimore, Maryland; Johns Hopkins Bayview Medical Center, Baltimore, Maryland; Johns Hopkins University School of Medicine, Baltimore, Maryland; Johns Hopkins Bayview Medical Center, Baltimore, Maryland; Johns Hopkins Bayview Medical Center, Baltimore, Maryland; Johns Hopkins Bayview Medical Center, Baltimore, Maryland; Johns Hopkins Bayview Medical Center, Baltimore, Maryland; Johns Hopkins Bayview Medical Center, Baltimore, Maryland; Johns Hopkins University School of Medicine, Baltimore, Maryland; Johns Hopkins Bayview Medical Center, Baltimore, Maryland; Johns Hopkins Bayview Medical Center, Baltimore, Maryland; Johns Hopkins Bayview Medical Center, Baltimore, Maryland; Johns Hopkins Bayview Medical Center, Baltimore, Maryland; Johns Hopkins Bayview Medical Center, Baltimore, Maryland; Johns Hopkins University School of Medicine, Baltimore, Maryland; Johns Hopkins Bayview Medical Center, Baltimore, Maryland; Johns Hopkins Bayview Medical Center, Baltimore, Maryland; Johns Hopkins Bayview Medical Center, Baltimore, Maryland; Johns Hopkins Bayview Medical Center, Baltimore, Maryland; Johns Hopkins Bayview Medical Center, Baltimore, Maryland; Johns Hopkins University School of Medicine, Baltimore, Maryland; Johns Hopkins Bayview Medical Center, Baltimore, Maryland; Johns Hopkins Bayview Medical Center, Baltimore, Maryland; Johns Hopkins Bayview Medical Center, Baltimore, Maryland

## Abstract

**Introduction:**

Successful rehabilitation of patients with burn injuries requires an in-depth occupational therapy (OT) and physical therapy (PT) evaluation early in the acute phase of a patient’s injury to develop a detailed therapy plan of care. In addition, it is a standard of the American Burn Association for verified burn centers to have a comprehensive rehabilitation program designed for burn patients within 24 hours of admission. However, there is no standardized report for tracking timeliness of issuing PT/OT orders or completion of therapy evaluations by PT/OT after a patient is admitted to a burn unit. This quality improvement study details the creation of a “Time to PT/OT Order Placement and Evaluation Completion Report” and results of a quality improvement process used to improve these metrics.

**Methods:**

A multi-disciplinary workgroup was created and included a burn surgeon, PT and program nurse coordinator and an electronic report was created in collaboration with the hospital’s business intelligence team. The report included the following metrics: average time from admission to PT/OT order placement, average time from PT/OT order placement to therapist acknowledgement, and average time from PT/OT order placement to therapist completion of initial evaluation. A baseline report was run (3-months) and results were analyzed. A quality improvement process was then put into place including education of burn attending physicians, fellows, and rotating residents on timeliness of therapy order placement, therapy staff education on findings and a new process for morning coordination of burn therapist caseloads to prioritize new evaluations, and initiation of monthly reporting at the burn joint practice committee meetings. The report was then run for an additional 3-months and results were analyzed.

**Results:**

Baseline and post-quality improvement process results (3-month averages) were as follows: Average time from admission to PT/OT order placement increased from 3.4 to 8.3 hrs, average time from PT/OT order placement to therapist acknowledgement decreased from 43.2 to 31.5 hrs, and average time from PT/OT order placement to therapist completion of evaluation decreased from 43.6 to 35.1 hrs.

**Conclusions:**

After development of this report findings showed that the quality improvement process used to improve timeliness of inpatient PT/OT initial evaluation completion was effective. In addition, this report has been useful for metric reporting during burn joint practice committee meetings, the ABA verification process and burn therapy staffing/daily caseload management.

**Applicability of Research to Practice:**

This report could be used by other burn centers to help track and improve timeliness of initial PT/OT evaluations for acute burns. In addition, metrics created from this report could be used for the ABA verification process and help justify burn unit therapy staffing needs.

